# Assessment of the role played by domestic animals in jigger infection in Kandara sub-county, Kenya (case control study)

**DOI:** 10.11604/pamj.2021.39.231.25106

**Published:** 2021-08-10

**Authors:** Anthony Kiragu Gitau, Florence Awino Oyieke, Wolfgang Richard Mukabana

**Affiliations:** 1 School of Biological Sciences, University of Nairobi, Nairobi, Kenya

**Keywords:** Tungiasis, domestic animals, *Tunga penetrans*, infection, associated health problems

## Abstract

**Introduction:**

tungiasis is an ectoparasitosis caused by penetration of female sand flea, Tunga penetrans, into the skin of the susceptible animal and the consequent hypertrophy of the parasite. The objective of this study was to assess the association between domestic animals and jigger infection among the residents of Kandara sub-county in central Kenya.

**Methods:**

this was a case-control study that involved 776 individuals. Half of this number entailed case group who were jigger infected while the other half was the control, composed of jigger free participants. Structured questionnaires were, administered among the heads of the households to which the participants belonged to gather information concerning the animals they kept. Univariate analysis was, applied.

**Results:**

in this study, there were significant differences in age (P=0.008) between the two groups. Disparities in source of income (P<0.001) and level of education (P<0.001) came out as very significant factors in jigger infection. The case group was 10 times more likely to keep dogs than the control(9.6; 95% CI, 5.9-15.6). Case group was also 7 times more likely to rear chicken in comparison to the control (6.6; 95%, 4.2-10.4). The case group was 12 times more likely to let dogs loose in the compound in comparison to the control (12.1: 95%, 5.9-24.5). When compared to the control, this group was also 17 times more likely to keep chicken inside their houses (16.7: 95% CI, 6.8-35.9). **Conclusion:** there is a very high association between domestic animals and occurrence of tungiasis in Kandara sub-county.

## Introduction

Tungiasis (jigger infection) is a zoonotic ailment that results from infection of human and animals by sand flea (*Tunga penetrans*) [1,2]. The flea is commonly referred to as jigger or chigoe flea. Other African names for the flea include Nigua, Funza and Ndutu among others. Sand flea is reported to have been introduced into Africa from Brazil in sand ballast in 1872 [3]. It then spread to almost every African country between 17^th^ and 19^th^ centuries. The spread was facilitated by trade and war expeditions [4]. Jigger infestation is associated with a myriad of morbidities like ulcers, hyperkeratosis, auto amputation of digits, distorted gait among others [5]. If left unattended for a long time, tungiasis can debilitate the victim [5]. Associated health problems include anaemia and tetanus [6]. At the point of flea entry, many pathogenic bacteria have been detected: Wolbachia, *Clostridium tetani, Staphylococcus epidermidis*, proteus and Klebsiella species among others [7]. Tungiasis can facilitate in transmission of blood-oriented diseases e.g hepatitis B and HIV if non-sterilized instruments are used to extract the embedded flea [7]. The problem is worsened if these instruments are shared among family members [8].

The involved hosts cut across many animal categories. Among the primates, baboons and humans are largely infected [9]. Among the Rodentia, guinea pig, mice, rats and porcupine are, greatly infected [9]. Among the carnivores, dogs, cats and jaguar are the chief victims. In the order cingulata (armadillos), the nine-banded, big hairly and southern long nosed armadillos are highly infected [10]. The Artiodactyla (even-toed ungulates) also play hosts to *Tunga penetrans* [11]. They include cows, goats, pigs, sheep, camel, antelopes among others [12]. Among the birds, chicken are the leading hosts [13]. Jigger infected people can hardly progress economically [14]. This is because jigger menace affects almost every aspect of life including children´s education and social status of the infected people [15]. Many a time it has become difficult to control tungiasis and the accompanying health problems due to the involved stigma and the social-cultural beliefs inherent among jigger infected communities [16]. Domestic animals, in most infested homes, are usually set free to roam in the compound where they easily interact with both wild and peri-domestic animals thus facilitating the spread of the sand flea [17]. Thus, animals exacerbate jigger problem in endemic communities increasing the risk of human infection [18]. The importance of each animal species in human tungiasis epidemiology differs from one endemic area to the other [19]. While in West Africa pigs seem to be the chief reservoirs, in Brazil cats, dogs, and rodents happen to be the frequently infected hosts [20].

Although economic importance of tungiasis in animal production has not been analytically determined, documented literature depicts significant effects on growth rate, defects of limbs and secondary bacterial infections [21]. Tungiasis has been reported to cause agalactia in lactating sows with ensuing starvation of piglets if it affects their mammary glands [22]. Poor production and low market value of these products contribute enormously to the rise of poverty at community level [22]. Most jigger-infected communities do not know the role played by domestic animals in exacerbating tungiasis and thus freely interact with these animals oblivious of the exposure [23]. This study assessed and compared the kind of domestic animals kept by both jigger infected and jigger free people in Kandara sub-county. The sole aim was to evaluate the role these animals may have played in the spread of the ectoparasitosis in the area. Jigger health problem has become perennial in Kandara in spite of the government and other stake holders intervening with chemicals time and again. The results will be vital in facilitating all rounded future interventional measures, which will lender most jigger endemic areas free of the disease.

## Methods

**Study area and population:** this research was, carried out in Kandara sub-county in Muranga. Muranga County is among the jigger endemic areas in Kenya and thus Kandara served as a representative of all the sub-counties in the region. Kandara sub-county is about 60 km from, the capital city of Kenya, Nairobi. Muranga County has two rainy seasons, with the rain ranging between 1200 and 1600 mm. The temperature ranges between 21 and 35°c. The region has a volcanic loam soil favorable for jigger flea multiplication. Kandara sub-county is exactly located at latitude 0°54´0S and longitude 37°0´0 E. The altitude is 5308 feet above the sea level. The sub-county has an area of approximately 236 sq km and a population of about 175098 people, according to 2019 national census. The resident community is, characterized by unemployment, overcrowding, and dilapidated housing mostly of mud walls. Illiteracy and poor hygienic conditions are also rampant. According to an observation made in prior visits to the study area, a good number of children remain at home during school days.

**Ethical clearance:** this study was, cleared by Kenyatta National Hospital & University of Nairobi ethics committee (KNH/UoN) and licensed by National Commission of Science, Technology and Innovation (NACOSTI).

**Community entry:** this study was conducted between August and December 2019, a dry season when jigger infestation is normally on rise in Kenya. The study commenced with familiarization of the study team with the study area and population. Permission was first, sought from Muranga county director for health and administrative leaders like chiefs and their assistants. This was, preceded by submission of ethical approval and permit copies to county administrators. Assistance of the area public health officer and the community health workers was, sought at the beginning of the study to help identify the people and homesteads with jigger infection.

**Pretesting of data collection instruments:** the questionnaires and data sheets for the study were pretested in a pilot study conducted in the same area. This helped to get feedback from the research subjects that would aid in improving the main study.

**Study design and data collection:** a case-control study design was, used in this research. The survey was, conducted to find out the kind of domestic animals they keep, the ectoparasites they find on these animals, the kind of parasite control measures they apply and the span of time that elapses before the next control. Probation was also, done concerning where they house these animals and the extent to which they restrict them in movement. A specially structured questionnaire was, administered to both the case and control groups, which they filled with the help of community health extension workers.

**Consenting:** participants were requested to sign consent forms after voluntarily accepting to be involved in the study. Those who did not understand English were requested to fill a Gikuyu (local language) version of the consent form. For very young children, those very old and the mentally handicapped, their respective guardians signed the forms. For the illiterate and those unable to write, a witness of their choice was, called in to assist.

**Inclusion and exclusion criteria:** all kind of people qualified for this study, including children, the very aged, illiterate and mentally handicapped. This is because the assistance of guardians and witnesses was, sought in case of children and other unique cases. Age was not a factor, as no substance or chemical was to be, applied on the victims. People who had not lived in the study area for the last three months were however excluded from the study. This was because this group may have immigrated to the study area while already infected, and thus the prevailing risk factors may not have been the cause.

**Determination of sample size and the sampling method:** a case- control study sampling design was employed to determine sample size. The sample size was calculated using Epi Info, a program developed by Centers for Disease Control (CDC). Sample size calculation with a 1: 1 ratio between case and control, to give results with 95% confidence limits, an assumed prevalence of exposure of 40% among controls, 80% power and least extreme odds' ratio to be detected of 1.5, indicated the necessity for a sample of 776 participants (388 cases and 388 controls).

**Case group and control recruitment:** to allow for loss that could follow-up, the number was rounded off to 800. The victims were, randomly picked from the six-kandara wards, with the assistance of Kandara Sub-county public health officer and his community health extension workers (CHEWS). Jigger control campaigns are common in Kandara sub-county and thus every CHEW has a data of all jigger infested homesteads in his respective ward. From this data base, 67 jigger infected individuals were randomly picked from each ward using randomly generated numbers. According to the 2019 national census, the average number of occupants per household in Kandara sub-county is 5. Though every infested person in the household was recommended for treatment at Kandara hospital, only 1 victim per household was picked for the study. This helped to include as many households as possible. From the immediate neighborhood of each jigger infested home, five jigger free homesteads were earmarked for control selection and assigned some random numbers. From a pool of these five, one homestead was randomly picked to be part of the control. Thus, every ward also produced 67 homesteads for the control (ratio of 1: 1). Both the heads of the case group and control households were requested to fill specially designed questionnaires for the study.

**Data analysis:** data was entered into an excel database where errors were checked and coding done. It was, then exported to SPSS (23.0) for a univariate analysis between the case group and the control. Odds ratio together with their 95% confidence intervals (CI) and P-values were, determined. Odds ratio were determined using 2 x 2 contingency tables. This analysis was to establish the significance of the differences in the various animal parameters (explained in the design) between the case group and the control.

## Results

**Social-demographic characteristics:** in this study, the age difference between the control and the case group individuals was significant (P = 0.008). Gender difference was also significant with males featuring more in the case group (53.3%) than female (46.7%). Among the control participants, female were 57.1% while the male were 42.9%. In both the control and the case groups, majority of the participants were married (53.3% and 75% respectively). Majority among the case group individuals (81.5%) had their monthly income below 50US$, while half of the control (50%) had an income of between 50 and 100 US$. Thirty-three-point seven percent (33.7%) of the control earned above 100US$. Most of the participants in the case group were casual laborers (57.6%). Twenty-four-point five percent (24.5%) of them however, received help from relatives. Majority among the control were self- employed (46.4%). Thirty three percent of them however were casual laborers (Table 1). Religious differences between the control and the case group were not significant (P = 0.41) as most of them in the two groups were predominantly Christians. More than a half of the case group (51.9%) did not have formal education at all. Most of the control participants (70% and above) had at least attained the primary level of formal education (Table 1).

**Table 1:** socio-demographic characteristics of the study participants

Variable	Case number and % (n=388)	Control number and % (n=388)	P-value
**Age in years**			
<30	36 (8.7)	14 (2.7)	0.008
31-40	68 (17.4)	100 (26.1)	
41-50	46 (22.3)	114 (29.9)	
51-60	96 (25.0)	68 (17.4)	
>60	102 (26.6)	92 (23.9)	
Gender			
Male	206 (53.3)	168 (42.9)	0.047
Female	182 (46.7)	220 (57.1)	
**Marital status**			
Married	200 (53.3)	280 (75.0)	<0.001
Single	96 (25.0)	34 (8.2)	
Widow	54 (13.6)	48 (12.5)	
Widower	30 (7.1)	12 (2.2)	
Divorced	8 (1.1)	12 (2.2)	
**Income per month (KES)**			
<5000	306 (81.5)	67 (16.3)	<0.001
5000-10000	67 (16.3)	190 (50.0)	
>10000	15 (2.2)	131 (33.7)	
Source of income			
**Formerly employed**	8 (1.1)	17 (3.8)	<0.001
Self employed	23 (5.4)	173 (46.4)	
Casual laborer	216 (57.6)	125 (33.3)	
Elderly fund	43 (10.9)	26 (6.0)	
Help from relatives	93 (24.5)	41 (10.4)	
Others	5 (0.5)	6	
**Education**			
None	195 (51.9)	87 (22.3)	<0.001
Primary and below	151 (39.9)	143 (37.5)	
Secondary	31 (7.7)	111 (28.8)	
Tertiary	11 (0.5)	47 (10.9)	
**Religion**			
Christian	361 (96.7)	367 (97.8)	0.410
Muslim	7 (0.5)	9 (0.5)	
Traditionalist	11 (1.6)	0	
None	9(1.1)	12(1.6)	

**Univariate analysis of domestic animals related factors:** in this study, there were, significant differences in the keeping of animals between the case group and the control: cows (p<0.001), dogs (p<0.001) and chicken (p<0.001). There were also significant differences between the two groups concerning where they keep these animals (p<0.001 in each case). Significant difference also exists between the case group and the control in the manner they restrict the animal's movements (p<0.001), and the way they allow them to interact with other animals in the neighborhood (p<0.001). A significant difference also features in the level of animal infection by fleas between the two groups (p<0.001) and in the control of the ectoparasite(p<0.001). Significant difference also occurs in the manner these two groups apply these control measures (p<0.001). Sixty five percent of the case group employs, the parasite control measure after a period exceeding one month, while 59% of the control does it at least once per month (Table 2).

**Table 2:** univariate analysis of domestic animals related factors

Variables			Case	Control	OR (95%CI)	P-value
Which domestic animals do you have in the homestead?	Cow	Yes	62 (33.7)	100 (54.3)	0.4 (0.3-0.7)	<0.001
		No	122 (66.3)	84 (45.7)	1.0	
	Goats	Yes	54 (29.3)	56 (30.4)	0.9 (0.9-1.5)	0.820
		No	130 (70.6)	128 (69.6)	1.0	
	Dog	Yes	150 (81.5)	58 (31.5)	9.6 (5.9-15.6)	<0.001
		No	34 (18.65)	126 (68.5)	1.0	
	Chicken	Yes	140 (76.1)	60 (32.6)	6.6 (4.2-10.4)	<0.001
		No	44 (23.9)	124 (67.4)	1.0	
	Cat	Yes	20 (10.9)	26 (14.1)	0.7 (0.4-1.4)	0.344
		No	164 (89.1)	158 (85.9)	1.0	
	Other	Yes	14 (7.6)	19 (10.3)	0.7 (0.3-1.5)	0.362
		No	170 (92.4)	165 (89.7)	1.0	
Where do you keep these animals?	Cow	House, we live	-	-		
		Compound	50 (80.7)	32 (32.0)	8.9 (4.2-18.9)	<0.001
		Their house	12 (19.3)	68 (68.0)	1.0	
	Goats	House, we live	19 (35.2)	9 (16.1)	4.2 (1.6-10.7)	0.003
		Compound	14 (25.9)	6 (10.7)	4.6 (1.5-13.6)	0.005
		Their house	21 (38.9)	41 (73.2)	1.0	
	Dog	House, we live	-	-		
		Compound	125 (83.3)	17 (29.3)	12.1 (5.9-24.5)	<0.001
		Their house	25 (16.7)	41 (70.7)	1.0	
	Chicken	House, we live	99 (70.7)	8 (13.3)	16.7 (6.8-35.9)	<0.001
		Compound	-	-		
		Their house	41 (29.3)	52 (86.7)	1.0	
	Cat	House, we live	20 (100.0)	26 (100.0)	-	-
		Compound	-	-		
		Their house	-	-		
	Other	House, we live	2 (14.3)	3 (15.8)	1.1 (0.1-8.7)	0.920
		Compound	6 (42.9)	6 (31.6)	1.7 (0.4-7.6)	0.508
		Their house	6 (42.9)	10 (52.6)	1.0	
Do you restrict these animals in movement?						
Yes			69 (37.5)	112 (60.9)	0.4 (0.3-0.6)	<0.001
No			115 (62.5)3	72 (39.1)	1.0	
Do these animals interact with other animals in the neighborhood?						
Yes			115 (62.5)	70 (38.0)	2.7 (1.8-4.1)	<0.001
No			69 (37.5)	114 (62.0)	1.0	
Which ectoparasites have you seen on your animals?						
None			12 (6.5)	100 (54.3)	1.0	
Fleas			78 (42.4)	34 (18.5)	19.1 (9.2-39.3)	<0.001
Ticks			43 (23.4)	16 (8.7)	22.4 (9.8-51.3)	<0.001
Fleas and ticks			51 (27.7)	34 (18.5)	12.5 (6.0-26.2)	<0.001
Do you control these ectoparasites?						
Yes			62 (33.7)	101 (54.9)	0.4 (0.3-0.6)	<0.001
No			122 (66.3)	83 (45.1)	1.0	
Which control measures do you employ (if yes )						
Spraying with chemicals			10 (16.1)	90 (89.1)	0.1 (0.0-0.4)	<0.001
Removing manually			43 (69.4)	1 (1.0)	47.8 (5.4-421.6)	<0.001
Washing their houses			9 (14.5)	10 (9.9)	1.0	
How often do you effect these control measures?						
Daily			-	-	-	
At least once per week			1 (1.6)	2 (2.0)	0.3 (0.0-3.1)	0.256
At least once per two weeks			1 (1.6)	4 (4.0)	0.1 (0.0-1.3)	0.043
At least once per three weeks			7 (11.3)	14 (13.9)	0.3 (0.1-0.8)	0.001
At least once per month			13 (21.0)	60 (59.4)	0.1 (0.0-0.2)	<0.001
Other (after a period exceeding 1 month)			40 (64.5)	21 (20.8)	1.0	

## Discussion

In this survey, the highest percentage of participants in the case group was of 60 years old and above. The highest percentage among the control group was in middle class (41-60 years age group). Marked increase in infestation in the old people (60 years and above) is caused by other factors besides their normally compromised immune system. It is, attributed to behavior with age and different exposure [24]. Middle-aged groups, for example, largely consist of the working people who spend most of the time away from the community and may have diverse disease related behaviors; they do thorough inspection and extract embedded fleas more scrupulously [25]. In this study, marital status was not significant, with most of the control and case group participants being in the married category. Gender difference was however significant, with most of the jigger infested household heads being males. Whereas in some studies males have been depicted to be more prone to jigger infection than female, some have found females being more vulnerable or no gender difference at all in infestation [26]. In this study, poverty featured prominently as a factor exacerbating jigger infection, with most of the infested household heads being casual laborers and earning below 50US $ per month. Majority of the control individuals were self-employed, with most of them (above 75%) earning more than 50US $ per month. This concurred with studies conducted by many researchers on poverty as a factor aggravating the ectoparasitosis [27]. More than half of the jigger infested household heads did not have formal education at all. Above 70% of the control, household heads had at least attained the primary level of formal education. This concurred with a study conducted by Kimani, Nyagero and Ikamari in 2012 where they reported that jigger infected people are normally from meager educational background [28]. In other studies, disability and other forms of health problems associated with jigger infection have been reported to hinder acquisition of formal education [29]. In this study, religious differences did not feature out as a factor influencing jigger infection. In another study, however, there are, some sects that have been, reported to view parasites-like jigger flea-as organisms of equal rights to man before God and thus tampering with their lives would result in sin. Such sects do not present their health problems to hospitals [30].

**Figure 1:**
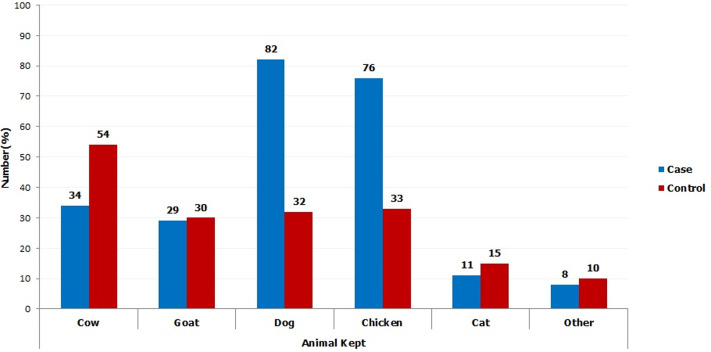
graphic comparison between the domestic animals kept by the case group and the control; jigger infested participants kept chicken and dogs in large numbers, which are mainly major hosts of jigger flea

**Figure 2:**
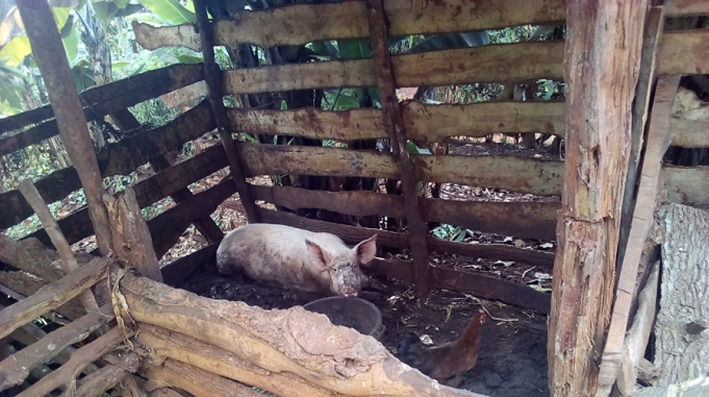
demonstration of the freedom the animals among jigger-infested people in Kandara are given to interact; the poor pigsty can allow both domestic and peridomestic animals like rats to enter at will

In this study, dog featured out as the most common animal kept by the jigger infested participants (Figure 1). A very large percent of them allows the dogs to, freely, roam in the compound, creating a chance for them to easily interact with wild and domestic animals from the neighborhoods. Thus, dogs appear to be very significant in the epidemiology of jigger flea in Kandara sub-county. These findings agree with other studies conducted in Brazil and Nigeria where dogs have been, reported to be main reservoir hosts in tungiasis. It also concurs with another study conducted in rural Uganda by Mutembi and others in 2015 where they reported that dogs are normally, not restrained in most villages where tungiasis is highly prevalent [31]. It however disputes their outcome where they concluded that dogs are not very important in the ectoparasitosis epidemiology simply because they were comparatively not many in their jigger infested study area [32]. Chicken were the second most common animals reared in the jigger-infested homesteads in the study area (Figure 1). As it was, found out, these fowls mainly spend the night in the same houses as the people. During the day, the birds are, allowed to roam in the compound to forage for food and thus facilitating their interaction with other animals (Figure 2). Chicken thus stands out as major hosts in spreading jigger flea in Kandara sub-county. Studies conducted by other researchers have depicted domestic animals, including chicken, as important reservoirs of *Tunga penetrans* and significant in human infection [33]. Studies conducted in Brazil and Tanzania have reported that sharing the same dwellings with animals establishes a potential basis of infection among family members [34]. This risk is especially enforced by the fact that many people are not informed that animals are also, infected by jigger flea and that living closely to them could facilitate infection [35]. Chicken and other aves are reported to be potential hosts for *Tunga penetrans* [36].

**Figure 3:**
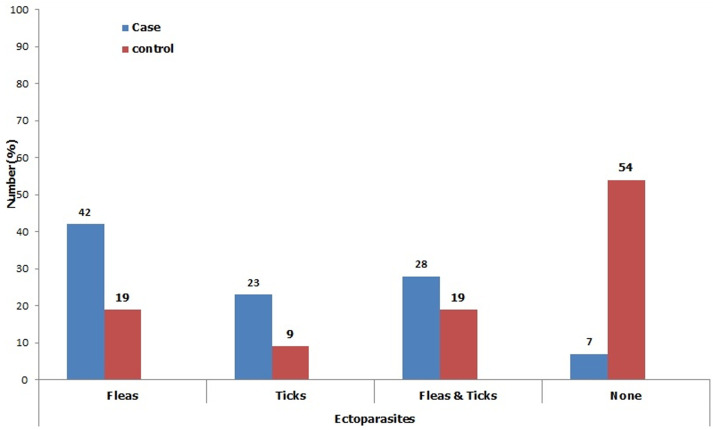
depiction of the kinds of ectoparasites found on the animals kept by the case group and control individuals; most of the jigger-infested participants indicated that fleas are common ectoparasites on their animals

**Figure 4:**
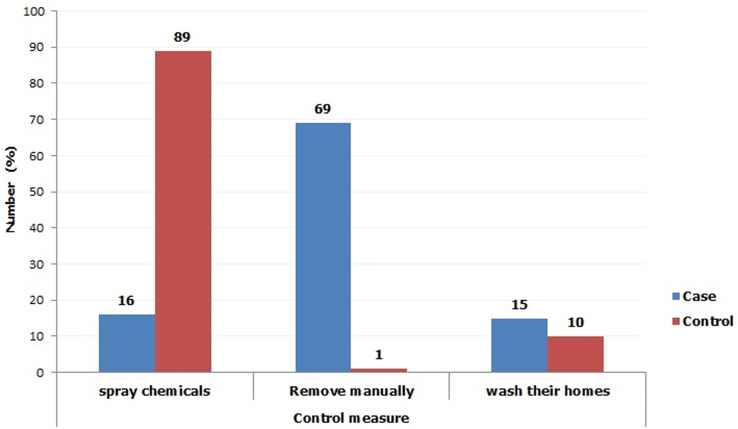
demonstration of the ectoparasites control measures between the case group and the control; most jigger free participants apply chemicals (which are, known to kill fleas) as opposed to jigger-infested individuals who control by removing them manually

Goats and cats did not feature out as important jigger reservoir hosts in this study. This concurred with the results of a study conducted in rural Ethiopia on animal reservoirs of *Tunga penetrans* where only a prevalence of 3.2% was, reported in goats [37]. Cats have been reported as important reservoir animals for tungiasis [38]. Although the case group, rear few cows in comparison to the control, they allow these animals to loiter in the compound as opposed to the later who largely practice zero grazing. Loitering animals facilitate the spread of the flea. Studies from South Africa depict cows as susceptible animals to *Tunga trimamillata* infection and *Tunga penetrans* as a co-infection [39]. The low number of cows among the case group was largely, attributed to poverty. Other animals encountered in this study include donkeys, pigs, ducks and doves; they all did not feature out as important reservoir hosts for jigger flea. In another study, however, pigs have been, exemplified as the most important reservoir for jigger flea [40]. Most of the jigger-infected participants indicated that fleas are a common ectoparasite on their animals (Figure 3). Most of the jigger free individuals observed that they barely find fleas on their animals. This was, corroborated by the fact that most of them regularly spray their animals with chemicals as a control measure. Chemicals normally used in jigger control interventions like propoxur insecticidal dust or spray, carbaryl insecticidal dust and cypermethrin spray have been reported to have high knockdown effect on jigger flea [40]. When it comes to controlling the ectoparasites, the jigger- infected participants remove the fleas manually after a period exceeding one month (Figure 4). Manual removal of fleas cannot help control tungiasis as some will remain hidden in the animal fur [40]. Again, effecting the control measure after a period exceeding one month will allow the flea to complete its life cycle and multiply immensely [41]. A control method with a high knock down effect and which targets both on host and off host stages of sand flea development is highly recommended in the ectoparasites eradication interventions.

## Conclusion

There is a high association between rearing of domestic animals and occurrence of tungiasis in Kandara sub-county. This relationship has been profoundly depicted in cases where dogs and chicken have been kept. The government and other involved stakeholders should carry out an educational campaign on the role played by domestic animals in tungiasis, alongside jigger control interventional measures for the eventual disease eradication to be attained. The government should start programs where human, animal and environmental friendly pesticides are regularly applied on domestic animals and their dwellings. This would help kill both the on- host and off-host stages of jigger flea.

### What is known about this topic

- Tungiasis is generally associated with resource poor people;
- Tungiasis is rampant among people of meager educational background.

### What this study adds

- Domestic animals may have a significant role in aggravating jigger infestation;
- Regular spraying of animals with chemicals may help reduce tungiasis.
